# Increased total plasma TGF-β1 levels and deregulated IgA-IgM axis in chronic obstructive pulmonary disease

**DOI:** 10.5937/jomb0-57461

**Published:** 2026-02-27

**Authors:** Rajna Minić, Olivera Đukić, Dejana Kosanović, Dejan Žujović, Aleksandra Ilić, Marko Panić, Brižita Đorđević

**Affiliations:** 1 Institute of Virology, Vaccines and Sera, "Torlak", Department of Protein Engineering and Biochemistry, Belgrade; 2 Faculty of Pharmacy, University of Belgrade, Department of Bromatology, Belgrade; 3 Institute for Medical Research - National Institute of the Republic of Serbia, University of Belgrade, Group of Immunology, Belgrade; 4 Municipal Institute for Lung Diseases and Tuberculosis, Belgrade; 5 Faculty of Medicine, University of Belgrade, Belgrade; 6 Institute of Virology, Vaccines and Sera, "Torlak", Department of mRNA, Belgrade

**Keywords:** chronic obstructive pulmonary disease, TGF-β1, IgA, IgM, Pseudomonas aeruginosa, hronična opstruktivna bolest pluća, TGF-β1, IgA, IgM, Pseudomonas aeruginosa

## Abstract

**Background:**

Chronic obstructive pulmonary disease (COPD) is characterised by obstruction of the small airways and includes "flare-ups", which are sudden and significant worsening of symptoms, sometimes caused by infections.

**Methods:**

Patients with COPD (n = 38) and acute bronchitis (AB, n=35) were grouped based on age and examined at two time points: during flare-ups/infections and at day 30. We measured various biomarkers, including total plasma TGF-<span style="color: rgb(32, 33, 34); font-family: sans-serif; font-size: 16px; background-color: rgb(248, 249, 250);">β</span>1 levels, total IgA, total IgM, and Pseudomonas aeruginosa-specific IgA levels.

**Results:**

Increased TGF-<span style="color: rgb(32, 33, 34); font-family: sans-serif; font-size: 16px; background-color: rgb(248, 249, 250);">β</span>1 levels were detected in COPD patients in comparison to acute bronchitis patients, with no significant change observed at day 30. Paired analysis revealed a significant decrease in total plasma IgA levels in COPD patients on day 30. No significant difference in specific P aeruginosa IgA levels was observed between the two patient groups. Interestingly, a correlation between total IgM and IgA levels was absent in COPD patients, unlike in those with acute bronchitis. While a positive correlation existed between age and IgA level in patients with acute bronchitis, this correlation was negative in patients with COPD. A significant correlation was observed between total IgM and P aeruginosa-specific IgA in acute bronchitis patients. On the other hand, COPD patients showed no correlation between total IgA or IgM and P aeruginosa-specific IgA levels at different time points.

**Conclusions:**

We report on the deregulated IgA-IgM axis in COPD and call for thorough, larger-scale studies of the humoral immune system in this pathology.

## Abstract (sr)

**Uvod:**

Hronična opstruktivna plucna bolest (HOBP) se ispoljava kao opstrukcija malih disajnih puteva i uključuje iznenadno i značajno pogoršanje simptoma, ponekad uzrokovano infekcijama.

**Metode:**

Pacijenti sa HOBP (n=38) i akutnim bronhitisom (AB, n=35) su grupisani na osnovu starosti, a plazma je uzorkovana u dve vremenske tačke: tokom pogoršanja/infekcije i nakon 30 dana. Mereni su različiti parametri, uključujući ukupne nivoe TGF-<span style="color: rgb(32, 33, 34); font-family: sans-serif; font-size: 16px; background-color: rgb(248, 249, 250);">β</span>1 u plazmi, ukupni IgA, ukupni IgM i nivoe IgA antitela specifičnih za Pseudomonas aeruginosa.

**Rezultati:**

Povećani nivoi TGF-<span style="color: rgb(32, 33, 34); font-family: sans-serif; font-size: 16px; background-color: rgb(248, 249, 250);">β</span>1 su otkriveni kod pacijenata sa HOBP u poređenju sa pacijentima sa akutnim bronhitisom, bez značajne promene nakon 30 dana. Značajno sniženje ukupnog nivoa IgA u plazmi primećeno je kod pacijenata sa HOBP tridesetog dana. Nije primećena značajna razlika u nivoima IgA antitela specifičnih za P aeruginosa između dve grupe pacijenata. Zanimljivo je da je korelacija između ukupnih nivoa IgM i IgA, koja je detektovana kod pacijenata sa akutnim bronhitisom, izostala kod pacijenata sa HOBP. Dok je pozitivna korelacija između starosti i nivoa IgA postojala kod pacijenata sa akutnim bronhitisom, kod pacijenata sa HOBP korelacija je bila negativna.

**Zaključak:**

Ovde izveštavamo o deregulisanoj IgA-IgM osi kod pacijenata sa HOBP i ukazujemo da postoji potreba za sprovođenjem opširnijih studija humoralnog dela imunskog sistema u ovoj patologiji.

## Introduction

Chronic obstructive pulmonary disease (COPD) is a progressive lung disease that causes breathingdifficulties due to obstruction of the airways, leading to reduced airflow, which induces coughing and wheezing in the chest. It is typically non-reversible or not fully reversible [1]. COPD is often caused by long-term exposure to irritants such as cigarette smoke [2], air pollution, dust and chemicals [3][4]. The symptoms of COPD, such as shortness of breath, cough, wheezing, and chest tightness, usually develop gradually and worsen over time, making it difficult for individuals to perform everyday activities. COPD can also lead to other complications such as respiratory infections, lung cancer [5], and heart disease [6]. In fact, COPD is a third leading cause of death according to World Health Organisation data collected in 2019 [7].

Exacerbations of COPD manifest as acute respiratory infections and bronchial asthma, often resulting in a decline in lung function and quality of life. One of the main goals of COPD therapy is to reduce the number and frequency of exacerbations, worsening or flare-ups. Current guidelines for the treatment of COPD [8] recommend, in addition to bronchodilators, the use of inhaled corticosteroids and phosphodiesterase inhibitors. Auxiliary treatment can include mucolytics, which play a crucial role in preventing exacerbations by enhancing mucociliary clearance [9].

Several genetic variants have been identified and associated with an increased risk of COPD. These variants are involved in processes such as inflammation, antioxidant defence, and lung development. Genetic factors alone are not sufficient to cause COPD, and smoking and environmental exposures remain the primary risk factors for the disease. One of the most well-known genetic risk factors for COPD is a variant in the alpha-1 antitrypsin (AAT) gene, which can lead to low levels of alpha-1 antitrypsin (AAT) protein in the blood and increased risk of COPD development, especially in individuals who smoke [10][11]. AAT is a protein produced by the liver that plays a crucial role in protecting the lungs and other organs from damage. However, it can also harm normal tissues if not properly regulated, underscoring the important role of the immune system in COPD [12].

According to Konigshoff et al. [13], the first association between the TGF-β1 genotype and increased susceptibility to COPD was reported in 2004 by Wu et al. [14]. It was found that the proline allele at codon 10 of the *TGFB1 *gene occurs more frequently in control subjects than in individuals with COPD. The same year, Celedon et al. [15], based on their findings, hypothesised that genetic variants in or near the *TGFB1 *gene influence the pathogenesis of COPD among cigarette smokers. Since then, numerous studies and debates have been published on the subject, keeping TGF-β in the spotlight in relation to COPD.

Literature data show that COPD patients with low circulatory IgA levels show a higher risk for exacerbations [16]. Downregulation of pIgR expression on bronchial epithelium was also detected in COPD patients [17][18][19]. The downregulation of pIgR was found to relate to airflow limitation and neutrophilic infiltration [19]. It seems that local IgA synthesis and production by B cells, which is upregulated in lung tissue [20] in COPD, does not lead to increased concentration of secretory IgA in bronchial secretions due to the defect in pIgR-mediated transepithelial transport [21]. Additionally, antigen-specific antibody responses are affected, as patients with severe COPD exhibit a defective IgA response against *Pseudomonas aeruginosa*, which may contribute to chronic/recurrent infections in these patients [22][23]. *P. aeruginosa* is a Gram-negative, aerobic bacterium commonly found in soil, water, and plant material. It is an opportunistic pathogen that can cause infections in humans, particularly in people with weakened immune systems, such as those with cystic fibrosis or those who have undergone organ transplants, but also in COPD patients. *P. aeruginosa* is known for its ability to form biofilms and become resistant to antibiotics and immune system defences, which makes it particularly difficult to treat infection [24]. In patients with COPD, *P. aeruginosa* infections can lead to worsening of symptoms, increased hospitalisation rate [25], and a decline in lung function. COPD patients from whom* P aeruginosa* can be identified in airway cultures have a markedly increased risk of exacerbations and death [26]. Treatment for *P aeruginosa* infections in patients with COPD typically involves the use of antibiotics. However, the choice of antibiotics may vary depending on the severity of the infection, the patient's overall health status, and the presence of antibiotic resistance. In some cases, intravenous antibiotics may be required, particularly for more severe infections. Additionally, *P aeruginosa* infections in COPD patients can be difficult to treat because the bacteria can develop resistance to antibiotics over time. Prevention of *P aeruginosa* infections in COPD patients involves avoiding exposure to the bacteria, particularly in healthcare settings.

To assess the interplay between the total IgA, IgM and *P aeruginosa* specific IgA levels with TGF-β1 levels, we have compared COPD patients with acute bronchitis (AB) patients at two time points: at disease flare-up for COPD patients, or at the beginning of the infection for AB patients, and upon a thirty-day interval.

## Materials and methods

### Study subjects

The study was approved by the Ethical Committee of the Clinical Centre of Serbia (Approval number: 832/25). The study included 38 subjects with an average age of 65 years (interquartile range, IQR 59-71) and a diagnosis of COPD. The patients were recruited during the exacerbation phase of the disease at the Clinic for Pulmonology, Clinical Centre of Serbia, in Belgrade, from November 2018 to May 2019. Exacerbation phase therapy included antibiotics (second-generation cephalosporins, quinolones, or macrolides) and corticosteroids when appropriate (methylprednisolone). Table 1

**Table 1:** Demographic characteristics of two patient populations. COPD - chronic obstructive pulmonary disease patients, AB - acute bronchitis patients.

	COPD<br>n=38	AB<br>n=35	*p*
Age, years<br>(IQR)	65 (59-71)	61 (48-69)	0.1691
Men/Women,<br>n (%)	27<br>(71)/11(29)	9(26)/26(74)	0.0001
Duration of<br>COPD, years	6.5 (2.7-10)	N/A	
GOLD stage,<br>n (%)	stage 2 - 18<br>(47)	N/A	
	stage 3 - 20<br>(53)	N/A	

Patients with acute bronchitis, with an average age of 61 years (interquartile range, 48-69 years), were clinically evaluated at the Municipal Institute for Lung Diseases and Tuberculosis in Belgrade (n=35). Patients were sampled from April to October 2018. Only patients with low CRP values were sampled for this analysis. The CRP values were 3.3±5.1 mg/L, and no bacterial pathogen was isolated. These patients were labelled as AB. Plasma was collected into 9 mL K_3_EDTA tubes, centrifuged for 15 minutes at 2,000 × g, and kept frozen until analysis.

### Plasma TGF-β1 determination

TGF-β1 levels were determined in plasma samples using the TGF-β1 Human/Mouse Uncoated ELISA Kit (Invitrogen; Thermo Fisher Scientific, Waltham, Massachusetts, USA) according to the manufacturer's recommendations.

### Determination of total plasma IgA concentration

Total plasma IgA levels were determined using a sandwich ELISA. Anti-human IgA1/IgA2, clone G20-359 (BioLegend), was used as the capture antibody at a concentration of 2 μg/mL. Plate coating was performed overnight at 4°C. Blocking was performed for 1 hour with 2% bovine serum albumin in phosphate-buffered saline (2% BSA/PBS). The quantification standard was pooled human serum, with total IgA concentration determined in a certified diagnostic laboratory using immunoturbidimetry against specific antisera (Aqualab, Belgrade). Washing was performed three times with PBS containing 0.05% Tween 20 (TPBS) and once with PBS alone. Sera were measured at a dilution of 1:1600, and for detection, HRP-conjugated AffiniPure Goat Anti-human IgA (Boster) was used at a concentration of 0.2 mg/μL.

### Determination of total plasma IgM concentration

Total plasma IgM levels were determined using a sandwich ELISA. The capture antibody, Anti-Human IgM (μ-chain specific) (B1265, Sigma-Aldrich), was used at a dilution of 1:500. Plate coating was performed overnight at 4°C. Blocking was done with 2% BSA in PBS for 1 hour. The quantification standard was pooled human serum, with total IgM concentration determined in a certified diagnostic laboratory using immunoturbidimetry against specific antisera (Aqualab, Belgrade). Washing was done three times with TPBS and once with PBS. Sera were diluted 1:16,000, and for detection, HRP-conjugated AffiniPure Goat Anti-Human IgM (Boster) was used at a concentration of 0.1 μg/mL.

### Determination of specific *P aeruginosa* IgA levels

In this study, *P aeruginosa* ATCC 6301 was used to determine the presence of specific anti-bacterial antibodies. The bacterium was propagated in Nutritious broth at 37°C. The procedure for coating Maxi-Sorp ELISA plates (Nunc, Thermo Fisher Scientific, Denmark) with microorganisms was carried out as previously described [27]. The ELISA was performed as described previously [28]. For the analysis of both bacteria-specific IgA and IgA subclasses, sera were diluted 1:100. The following monoclonal antibodies were used: anti-human IgA1/IgA2, clone G20-359 (Biolegend), anti-human IgA2 clone A9604D2 (Biolegend), both produced in mice and coupled to biotin and anti-human IgA1 alkaline phosphatase labelled, clone B3506B4 (Abcam). Biotinylated antibodies were further detected using streptavidin-alkaline phosphatase and 4-nitrophenyl phosphate.

### Statistical analysis

Chi-squared test was used for group comparison according to age and sex. The numeric data distribution was assessed for normality using the Shapiro-Wilk Test, given the relatively small group size, and was further visually examined. Data with a normal distribution were statistically evaluated using one-way ANOVA and Tukey's Multiple Comparison Test, or a paired t-test. When including data not showing normal distribution, the Kruskal-Wallis test with Dunn's post-hoc test, the Mann-Whitney U test, and the Wilcoxon matched pairs test were used. Correlations between individual results were performed with Spearman's rank test. Line art was exported from GraphPad Prism software (La Jolla, CA, USA).

## Results and discussion

**Figure 1:**
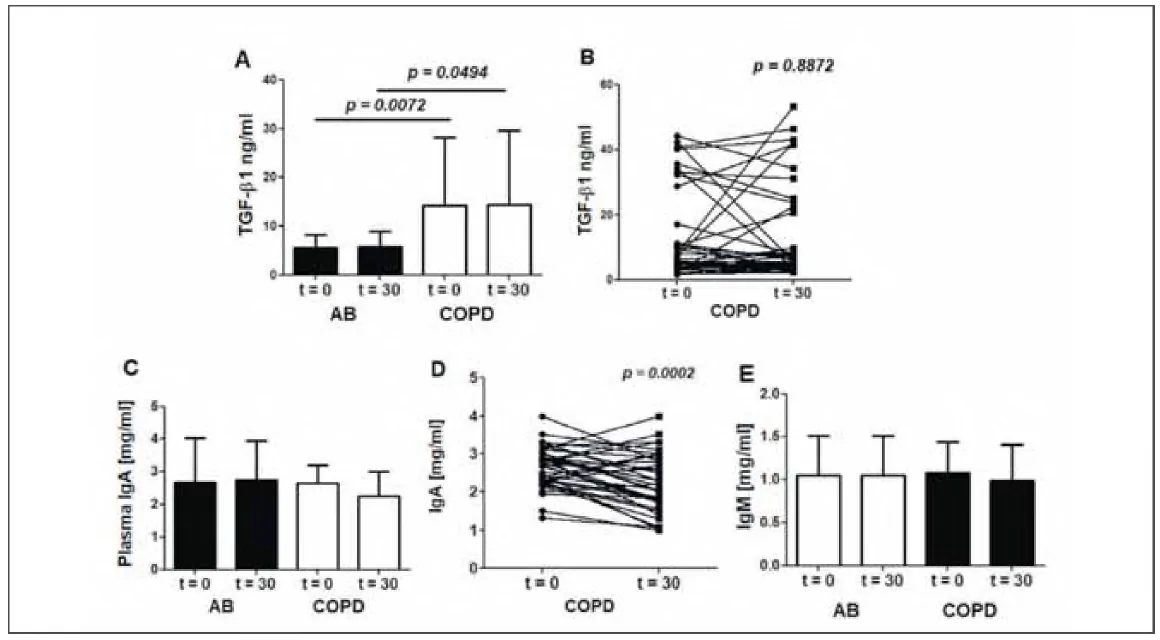
A) Plasma TGF-β1 levels in AB and COPD patient groups at t = 0 ad t = 30; B) Individual plasma TGF-β1 levels in COPD patient group at t = 0 and t = 30; C) Total plasma IgA levels in AB and COPD patient groups at t = 0 and t = 30; D) Individual plasma IgA levels in COPD patient group at t = 0 and t = 30; E) IgM levels in different patient groups at two time points. Mean values with standard deviations are shown. AB - acute bronchitis; COPD - chronic obstructive pulmonary disease; probability - p shown was calculated with the Mann-Whitney U test in A), the Wilcoxon matched pairs test in B), and with the paired t test in D).

Significantly higher level of TGF-b1 was detected in COPD patients compared to acute bronchitis patients (AB) at both time points (Figure 1A), which is consistent with existing literature data [29]. The data did not follow a normal distribution due to the presence of outliers, as shown in Figure 1B. No significant change was detected in AB patients over time, as the levels of TGF-β1 in the acute phase were no different from those at t=30 days. Additionally, there was no statistically significant change in TGF-β1 levels in COPD patients during the exacerbation phase compared to 30 days later on a group level (Figure 1A). However, the individual values of TGF-β1 in COPD patients show that, although TGF-β1 levels are relatively stable for some patients, changes were detected in others and were not uniform. While in some patients there was a decrease in TGF-β1 levels in others there was an increase, Figure 1B, which is in accordance with the fact that CORD is a complex and heterogeneous lung condition and a collective term for a broad spectrum of diseases involving both the large and small airways as well as the lung parenchyma, according to van Eden and Hogg [29].

TGF-β plays an essential role in the growth and development of cells, tissues, and organs, including the lungs. Literature data states that in individuals with CORD, changes in the levels or activity of TGF-β contribute to the development and progression of the disease [30]. TGF-β has been shown to play a key role in regulating the process of tissue repair and remodelling in the lungs. In response to injury or inflammation, TGF-β can promote the growth of new cells and the formation of scar tissue [31], which can lead to airway narrowing and reduced lung function in individuals with CORD [32]. Studies have suggested that increased TGF-β activity in the lungs may contribute to the development of emphysema, one of the main components of CORD, characterised by damage to the air sacs in the lungs [33]. TGF-β may also be involved in the development of chronic bronchitis, another component of CORD characterised by inflammation and narrowing of the airways. Therefore, targeting TGF-β signalling pathways is a potential therapeutic approach for CORD, as it may help reduce the tissue remodelling and inflammation that contribute to the disease. However, further research is needed to fully understand the role of TGF-β in CORD and its potential as a target for new therapeutic interventions.

We have detected lower total IgA levels in CORD patients compared to AB patients at t=30, but this difference was not statistically significant (Figure 1C). A significant reduction in IgA levels was observed over time in CORD patients, as shown in Figure 1D. The levels of total IgM did not differ over time in AB patients and were roughly equal to those in CORD patients, as shown in Figure 1E.

IgA plays a crucial role in the immune system's defence against infections, particularly in mucosal tissues such as the respiratory tract [34][35]. In individuals with CORD, changes in IgA levels were observed in both the blood and lungs, which can impact the immune response and increase susceptibility to respiratory infections. Some studies have suggested that individuals with CORD may have lower levels of IgA in their lungs, which can contribute to chronic inflammation and increased susceptibility to respiratory infections. An association was found between the reduced expression of IgA secretory component in airway epithelium, airflow obstruction and neutrophil infiltration in severe CORD [17].

However, further research is needed to fully understand the role of IgA in CORD and its potential impact on disease progression and outcomes. Monitoring IgA levels may be beneficial in certain instances, such as when evaluating the risk of respiratory infections in individuals with CORD. Still, it is not routinely recommended as part of CORD management.

*Pseudomonas aeruginosa* is a type of gram-negative bacteria that can cause respiratory infections. In patients with CORD, it can be isolated from up to 15% of individuals [36], particularly those with more severe disease or more frequent exacerbations [37]. The damaged airways and impaired immune response make it easier for bacteria like *P aeruginosa* to colonise and infect the respiratory tract. *P aeruginosa* infections in CORD can lead to exacerbations and increase the risk of disease progression.

Plasma levels of *P aeruginosa*-specific IgA, IgA1, and IgA2 were measured in both patient groups at two time points.

**Figure 2:**
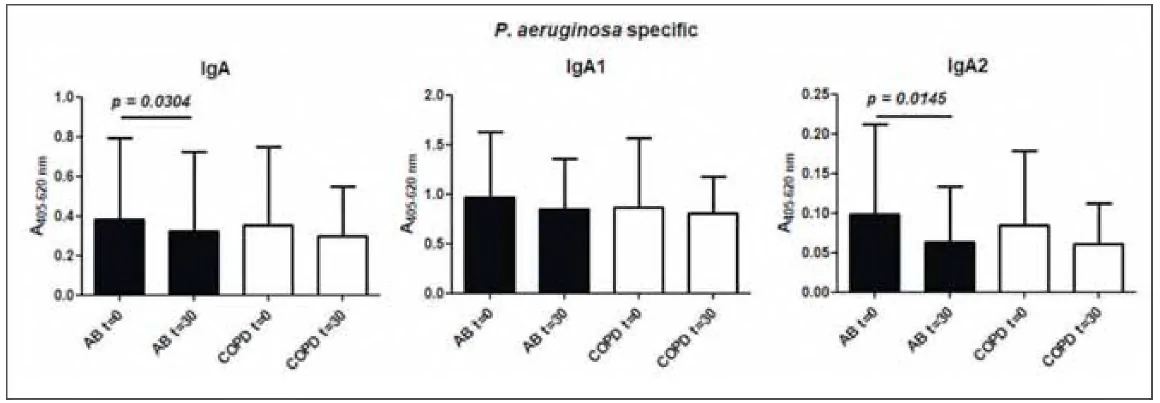
Plasma IgA, IgA1 and IgA2 levels specific for R aeruginosa in different patient groups at two time points. Mean values with standard deviations are shown. AB - acute bronchitis, COPD - chronic obstructive pulmonary disease. Probability - p values obtained with the Wilcoxon matched pairs test.

Large individual variations were observed, and no significant change in any of the levels was detected between the groups (Figure 2).

Preventing and managing* P aeruginosa* infections in COPD involves a combination of strategies, including reducing exposure to irritants and pollutants, optimising lung function, maintaining good respiratory hygiene, and using antibiotics judiciously. Vaccines against *P aeruginos*a are also being developed; unfortunately, so far, they have shown little clinical benefit [38][39]. Interestingly, the levels of P aeruginosa-specific IgA were no different from those in patients with acute bronchitis of the same age, however, we have measured plasma IgA levels as potentially relevant to the lung compartment. The possibility exists that this antibody class may not be crucial for vulnerability in COPD patients and specific IgG levels should also be evaluated.

**Figure 3:**
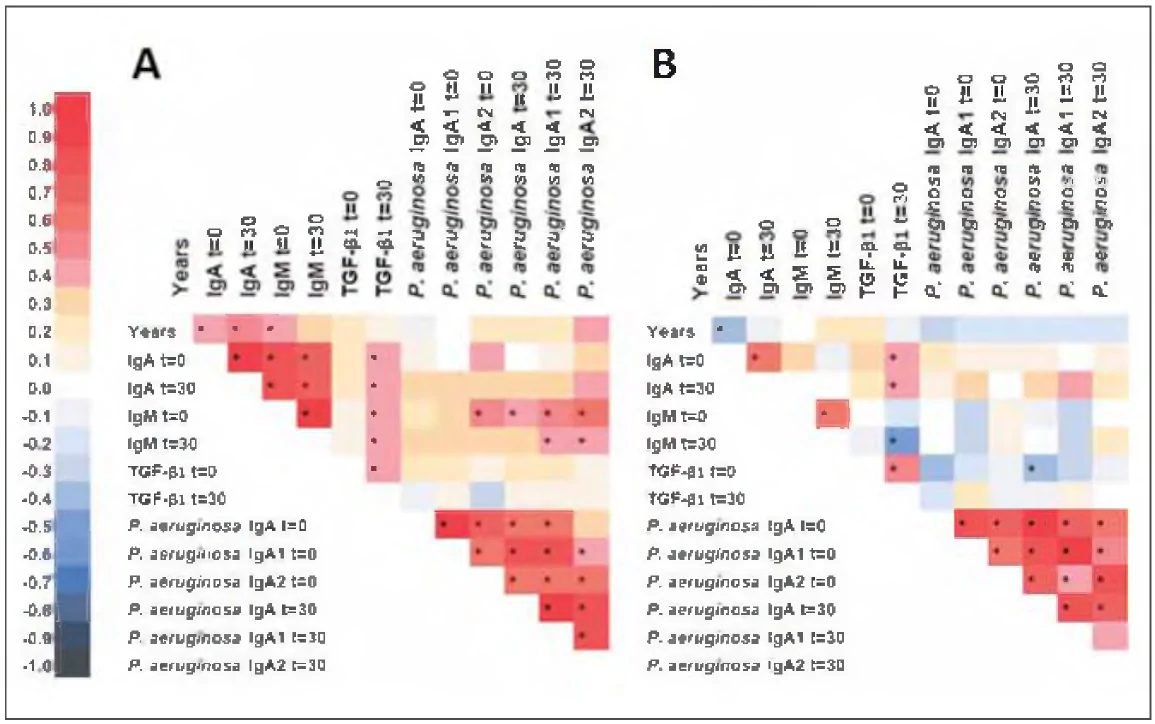
Graphic representation showing correlation coefficients obtained by using Spearman's rank test on different parameters in A) the acute bronchitis patients and in B) COPD patients. Numerical values representing Spearman's rank test coefficients were colour-coded according to the key shown on the left (red-positive, blue-negative). Statistical significance is denoted by p < 0.05, marked with an asterisk (*).

Correlation analysis of different parameters measured in AB and COPD patients is shown in Figure 3. A significant positive correlation was observed between the age of the patients and total IgA levels in AB patients, both at t=0 and at t=30 (Figure 3A). A significant positive correlation was also detected between patient age and total IgM levels at t=0 in AB patients (Figure 3A). In COPD patients, a significant negative correlation was observed between patient age and total IgA levels at t=0, which changed to some extent at t=30, where the correlation was no longer significant (Figure 3B).

In AB patients, total IgA levels at t=0 correlated positively with IgA at t=30, total IgM at both time points, and with TGF-β1 at t=30. Similar results were observed with total IgA at t=30 (Figure 3A). The fact that a significant positive correlation was observed in acute bronchitis patients between total IgA at t=0 and t=30, and between total plasma IgM levels at t=0 and t=30, indicates that these parameters represent relatively constant individual characteristics, unaffected by an episode of acute viral bronchitis.

In COPD patients, a significant positive correlation was observed between total IgA at t=0 and t=30, as well as between total plasma IgM at t=0 and t=30. This implies that these parameters represent relatively constant individual characteristics in COPD patients. Similarly, in COPD patients, TGFβ1 levels were highly correlated between the two time points, implying that, on a group level, this is also an individual characteristic. However, in COPD patients, there was no correlation between total IgA and IgM levels at either time point, which represents a difference between the two groups, suggesting the existence of a deregulated IgA-IgM axis in COPD patients.

In the case of P aeruginosa-specific total IgA levels and IgA isotypes, a high correlation was found in AB patients. A similar situation was observed in patients with COPD as well. However, as total IgM levels at both time points correlated significantly with *P aeruginosa*-specific IgA levels in AB patients, this correlation was absent in COPD patients.

Several studies support the conclusion that immune dysfunction leads to exacerbations and disease progression in COPD patients. Extensive immune dysfunction is attributed to the presence and functional activity of T regulatory cells, CD4+PD-1+ exhausted effector T cells and myeloid-derived sup+- pressor cells [39]. The high frequency of Foxp3^+^ Tregs demonstrated significant correlation with levels of TGF-β1 in patients with COPD. Thus, anti-bacterial immunity in patients with COPD is, according to Bhat et al. [40], limited by two critical factors: the inability of effector T cells to robustly respond to bacterial or viral antigens and the increased accumulation of functionally suppressive Tregs. The information on the humoral immunity in COPD patients, on the other hand, is relatively scarce. With this work, we aim to contribute by analysing components of humoral immunity at both absolute and specific levels, particularly in comparison to individuals diagnosed with acute bronchitis.

We acknowledge that this study had two drawbacks. First, as COPD is more frequent in males, we had a difference in patient sex, which could have, to some extent, influenced the results. We have previously measured significantly lower anti-pneumococcus (serotype 1) IgA1 titers in men in comparison to women, which was especially pronounced in men over 50 years of age [28]. Still, as this influence was not detected in total or specific antibody levels measured here, we think it is unlikely that it influenced the other parameters obtained. Second, the study had a limited number of patients; therefore, these measurements should be repeated on a larger number of patients and should include patient stratification. Additionally, this analysis should include a broader range of bacterial species, as well as an examination of different bacteria-specific antibody classes and subclasses. However, it is unclear whether this result will lead to clinical benefit for patients, particularly since older individuals generally exhibit a limited response to vaccination due to immunosenescence, the age-related decline in immune function.

A noteworthy result presented here is the decrease in total IgA levels with age in COPD patients, which is opposite to that observed in patients with acute bronchitis. We consider this result interesting, as although there is no clear correlation between plasma IgA levels and secreted IgA levels, in COPD, pIgR is often downregulated, leading to even lower IgA levels in secretions [21]. Furthermore, it has been shown that patients with COPD with low serum IgA levels show a higher risk for exacerbations [16].

Additionally, a positive correlation was detected between patient age and total IgM levels at t=0 in patients with acute bronchitis, but not in those with COPD. This, together with the fact that in COPD patients there is no correlation between total IgA and IgM levels at either timepoint, as well as the fact that in COPD patients there in no correlation between total IgM levels and *P aeruginosa* specific IgA levels implies that the perturbation in immune regulatory checkpoints exists in COPD patients evident at the level of humoral immunity and that it may be governed by or includes the IgA antibody class. Larger studies are needed to deepen the understanding of the influence of the humoral immune component on the pathology of COPD.

## Dodatak

### Acknowledgements

The authors would like to thank the patients for participating in this study. The authors would like to thank the healthcare staff at the Clinic for Pulmonology, Clinical Centre of Serbia, and the Municipal Institute for Lung Diseases and Tuberculosis. The authors thank Dr Vesna Ilić, Head of the Group of Immunology at the Institute for Medical Research, University of Belgrade, National Institute of the Republic of Serbia, for fruitful discussions.

### Funding

This work was supported by the Ministry of Science, Technological Development, andInnovations of the Republic of Serbia, Grant No 200177 (Contract No 451-03-136/2025-03/20177).

### Data availability statement

Data used in this study are available from the corresponding author upon request.

### Conflict of interest statement

All the authors declare that they have no conflict of interest in this work.
